# The immune-related symptom web in immune checkpoint inhibitor therapy: a longitudinal clinical safety surveillance framework for immune-related adverse events

**DOI:** 10.3389/fphar.2026.1942034

**Published:** 2026-09-04

**Authors:** Bing Li, Haina Che, Yanfeng Song

**Affiliations:** Department of Oncology, The First Bethune Hospital of Jilin University, Changchun, China

**Keywords:** active safety surveillance, corticosteroid stewardship, differential attribution, electronic symptom monitoring, immunotoxicity, patient-reported outcomes, pharmacovigilance, survivorship

## Abstract

Immune checkpoint inhibitors (ICIs) produce durable antitumor activity by releasing inhibitory immune pathways, but the same pharmacology can disrupt peripheral tolerance and generate immune-related adverse events (irAEs) that are delayed, recurrent, chronic, multisystem, and clinically nonspecific. Organ-specific guidelines are indispensable once toxicity is suspected; however, clinical surveillance commonly begins with an undifferentiated patient report or an objective abnormality rather than a confirmed organ diagnosis. This narrative review integrates clinical pharmacology, immunobiology, guidelines, pharmacovigilance principles, patient-reported outcome research, digital monitoring studies, and multidisciplinary implementation evidence. Focused, non-systematic searches of PubMed/MEDLINE and the Cochrane Library, supplemented by current guidelines and official regulatory sources, were finalized on 18 July 2026; representative strategies, an evidence-provenance map, and a structured comparison with existing approaches are provided in the Supplementary Material. We define an individual-level safety alert as a new symptom, functional change, laboratory abnormality, physiological change, imaging finding, or other clinically meaningful observation requiring contextual assessment. It is not equivalent to a formal population-level pharmacovigilance signal. The immune-related symptom web is a symptom-centered but not symptom-exclusive model that organizes exposure chronology, individual baseline, symptom and objective-data combinations, trajectories, competing diagnoses, corticosteroid tapering, discontinuation, rechallenge, and consequence-weighted urgency. The Signal-Attribution-Escalation-Longitudinal Management (SAELM) framework translates this information structure into an accountable workflow from alert capture to attribution, escalation, guideline-linked management, documentation, and reassessment. Its novelty lies in integrating and longitudinally organizing existing components rather than inventing each component. The Patient-Reported Outcomes version of the Common Terminology Criteria for Adverse Events (PRO-CTCAE) and electronic patient-reported outcome (ePRO) systems are positioned as capture and communication tools, not diagnostic or causal instruments. The models remain conceptual and hypothesis-generating. The most feasible first validation study is a prospective multicenter observational cohort with independent clinical adjudication; the primary safety outcome should be the false-negative proportion for severe or organ-threatening irAEs, accompanied by alert burden, response time, and potentially avoidable urgent escalation.

## 1 Introduction

Immune checkpoint inhibitors (ICIs) have altered the treatment of multiple solid tumors and hematological malignancies by blocking inhibitory pathways that restrain T-cell activation. Antibodies targeting cytotoxic T-lymphocyte antigen 4 (CTLA-4), programmed cell death 1 (PD-1), programmed death ligand 1 (PD-L1), and newer checkpoint combinations can restore antitumor immunity, but they also change the threshold for peripheral immune tolerance. The resulting immune-related adverse events (irAEs) differ from conventional dose-limited cytotoxic effects: they may affect almost any organ, involve more than one organ, recur during immunosuppressive treatment, persist chronically, or first become apparent after the last administered dose (Postow et al., 2018; Martins et al., 2019; Ramos-Casals et al., 2020; Schneider et al., 2021; Haanen et al., 2022).

Guidelines from the American Society of Clinical Oncology (ASCO), European Society for Medical Oncology (ESMO), Society for Immunotherapy of Cancer (SITC), and National Comprehensive Cancer Network (NCCN) provide essential organ-specific recommendations for investigation, severity assessment, treatment interruption, corticosteroid use, second-line immunomodulation, specialist referral, and rechallenge (Brahmer et al., 2021; Schneider et al., 2021; Haanen et al., 2022; Thompson et al., 2024). These algorithms generally become actionable after an organ syndrome is suspected. Between scheduled visits, however, the first warning may be a new cough, altered stool pattern, fatigue, rash, dizziness, weakness, palpitations, headache, visual change, or an imprecise report of feeling unwell. Alternatively, the first warning may be an asymptomatic laboratory, electrocardiographic, physiological, or imaging abnormality. None of these observations independently establishes immune-mediated causality.

The initial clinical task is therefore not simply to label an irAE. It is to recognize a potentially important change, preserve competing explanations, determine whether the pattern could represent rapidly progressive or irreversible disease, assign a response time, and ensure that responsibility for follow-up is explicit. Infection, cancer progression, thromboembolism, metabolic disturbance, comorbidity, chemotherapy, targeted therapy, radiotherapy, corticosteroid complications, and other medicines may produce the same symptoms or objective abnormalities. Over-attribution to ICIs and under-attribution are both potentially harmful.

Conventional post-marketing pharmacovigilance detects and evaluates aggregated drug-event associations, but spontaneous reporting is affected by under-reporting, stimulated reporting, duplicate reports, incomplete verification, and absent exposure denominators. A disproportionality signal does not establish incidence or individual causality (European Medicines Agency, 2017; U.S. Food and Drug Administration, 2026a; World Health Organization, 2026). Clinical safety surveillance operates at a different level: it captures and responds to changes in an individual patient. The present review connects these levels without conflating them.

We propose two related constructs. The immune-related symptom web is an information and interpretation model that connects exposure chronology, individual baseline, symptoms, objective findings, multisystem combinations, competing diagnoses, clinical actions, and response over time. The SAELM framework is an operational workflow that specifies how this information moves from capture to attribution, urgency assignment, management, documentation, and longitudinal reassessment. Both are conceptual and should complement rather than replace guideline-directed evaluation, Common Terminology Criteria for Adverse Events (CTCAE) grading, clinical judgment, or treatment decisions.

## 2 Methods

### 2.1 Review design

This article is a narrative review rather than a systematic review, scoping review, or meta-analysis. A narrative design was selected because the question spans mechanistic pharmacology, clinical guidelines, observational epidemiology, spontaneous-report pharmacovigilance, patient-reported outcomes, digital implementation, and multidisciplinary practice. These evidence streams use different units of analysis and were synthesized to develop an evidence-informed conceptual model rather than a pooled comparative estimate.

### 2.2 Information sources and search approach

PubMed/MEDLINE and the Cochrane Library were searched without a lower date limit, with the final focused update completed on 18 July 2026. Searches combined terms for ICIs and checkpoint targets; immunotoxicity and irAEs; symptoms, symptom clusters, networks, and trajectories; the Common Terminology Criteria for Adverse Events (CTCAE) and its Patient-Reported Outcomes version (PRO-CTCAE); patient-reported outcomes and electronic patient-reported outcome (ePRO) monitoring; pharmacovigilance, active surveillance, causality, and signal management; corticosteroids and steroid-refractory or steroid-dependent disease; rechallenge, delayed and chronic toxicity, survivorship, triage, workload, implementation, and equity. Organ-specific terms were added for gastrointestinal, pulmonary, hepatic, endocrine, cardiac, neurological, renal, dermatological, rheumatological, ocular, and hematological toxicities. Searches were supplemented by targeted consultation of ASCO, ESMO, SITC, NCCN, the National Cancer Institute, the U.S. Food and Drug Administration, the European Medicines Agency, and the World Health Organization and by backward citation searching. Representative strategies are reported in Supplementary Methods S1.

### 2.3 Eligibility, prioritization, and source selection

English-language human studies, guidelines, consensus statements, regulatory or pharmacovigilance documents, methodological publications, and selected translational studies were eligible. International guidelines and consensus statements were prioritized for management recommendations; systematic reviews, randomized trials, and cohorts were prioritized for comparative or prognostic claims; pharmacovigilance studies were used to characterize rare or reported high-consequence patterns; PRO/ePRO and implementation studies informed workflow design; and mechanistic studies were included when they helped explain nonspecific, multisystem, delayed, recurrent, chronic, or steroid-dependent presentations. Case reports were not used to support general incidence or treatment-effect claims. Titles, abstracts, and full texts were assessed iteratively for relevance to four organizing domains: pharmacological basis; temporal and multisystem phenotype; alert detection and attribution; and management and implementation.

### 2.4 Evidence provenance and framework development

During synthesis, statements were categorized as guideline-supported practice, empirical evidence from interventional or observational studies, evidence-informed interpretation, author-proposed framework elements, or future validation requirements. Candidate components of the symptom web and SAELM were identified from recurrent concepts across guidelines, pharmacovigilance standards, ePRO programs, remote triage models, multidisciplinary care, and literature on chronic, delayed, multisystem, and rechallenge-associated irAEs. Supplementary Tables S1–S4 summarize pharmacological and clinical determinants, sentinel alerts and escalation cues, organ-specific management principles, and staged validation priorities, respectively. Supplementary Table S5 links major claims and proposed components to representative sources, evidence type, limitations, and validation status, and Supplementary Table S6 provides a structured comparison with existing irAE guidelines, ePRO systems, triage pathways, symptom-network approaches, and conventional pharmacovigilance. Supplementary Table S5 is an evidence-provenance map, not a formal certainty-of-evidence assessment.

### 2.5 Reporting boundaries

SANRA was used to support transparency and scientific reasoning (Baethge et al., 2019), but it was not treated as a systematic-review method. No protocol was registered, no PRISMA flow diagram or formal screening log was produced, no duplicate independent screening was undertaken, and no study-level risk-of-bias tool was applied. The framework was not generated through Delphi consensus, formal model development, or patient co-design. These limitations preclude claims of exhaustive retrieval, formally graded evidence, or validated consensus.

## 3 Pharmacological and immunobiological basis

CTLA-4 and PD-1 regulate immune activation at partly distinct sites and stages. CTLA-4 constrains T-cell priming, particularly in lymphoid tissues, whereas PD-1 signaling limits effector activity in peripheral tissues and the tumor microenvironment. Therapeutic blockade can expand or reinvigorate antitumor T-cell responses, alter regulatory T-cell function, and change interactions among immune, stromal, and tumor cells. The same pharmacodynamic release of inhibitory control contributes to immunotoxicity (Postow et al., 2018; Ramos-Casals et al., 2020; Morad et al., 2021).

Population-level toxicity patterns differ across checkpoint targets and regimens, although they should not be treated as individual predictions. Anti-CTLA-4 therapy has often been associated with gastrointestinal, dermatological, and pituitary toxicity; PD-1/PD-L1 blockade has a broad peripheral-organ distribution; and dual-checkpoint blockade generally increases overall, severe, and multisystem toxicity compared with monotherapy. Tumor type, dose, treatment setting, follow-up, ascertainment, and co-administered therapy confound indirect comparisons (Khoja et al., 2017; Martins et al., 2019; Wang et al., 2018).

Approved ICIs are monoclonal antibodies with prolonged systemic persistence and time-varying or target-mediated clearance. Routine therapeutic drug monitoring is not established for irAE prevention because exposure-toxicity relationships are inconsistent and confounded by disease burden, albumin, body composition, inflammatory state, treatment response, and changing clearance (Sheng et al., 2017; Centanni et al., 2019; Geraud et al., 2021). The last dose is therefore necessary but insufficient temporal information. ICI class, treatment combination, cycle, interruption, discontinuation, immunosuppressive course, taper stage, and rechallenge are more useful surveillance variables than an assumed direct concentration-toxicity relationship.

irAEs probably arise through overlapping mechanisms, including expansion of autoreactive T-cell clones, reduction of regulatory constraints, cross-reactivity between tumor and healthy-tissue antigens, epitope spreading, cytokine dysregulation, B-cell activation, and autoantibody production. Tissue-resident immune populations and organ microenvironments may shape the affected organ, whereas pre-existing autoimmunity, organ comorbidity, human leukocyte antigen (HLA) variation, baseline immune states, and microbiome composition may modify susceptibility (Chaput et al., 2017). Most candidate predictors are associative and insufficiently validated for individual risk prediction (Berner et al., 2019; Das et al., 2018; Abdel-Wahab et al., 2018; Sung et al., 2023).

These mechanisms explain why early manifestations may be constitutional or multisystem rather than organ-specific, why cardiac–muscle–neuromuscular overlap can evolve rapidly, why endocrine destruction may be irreversible, and why inflammation may recur during corticosteroid tapering or after rechallenge. They also argue against relying on a single symptom or biomarker for attribution. Pharmacological and clinical determinants relevant to surveillance are summarized in Supplementary Table S1.

## 4 Temporal, multisystem, and chronic clinical phenotypes

The apparent epidemiology of irAEs depends on event definitions, follow-up, and ascertainment. Randomized trials provide comparative estimates but frequently exclude patients with complex comorbidity, autoimmune disease, or organ dysfunction. Observational cohorts include broader populations but are susceptible to referral and ascertainment bias. Spontaneous-report systems are valuable for rare and unexpected syndromes but cannot provide incidence because exposure denominators and reporting completeness are unknown (Salem et al., 2018; Wang et al., 2018; European Medicines Agency, 2017).

Temporal patterns are heterogeneous. Some dermatological, gastrointestinal, endocrine, pulmonary, neurological, cardiac, renal, or musculoskeletal events occur during early cycles; others develop after months, recur during immunosuppressive tapering, persist after acute inflammation resolves, or first become recognized after ICI discontinuation. Delayed and late-onset events have been described in cohorts and pharmacovigilance data, although prospective denominator-based evidence remains limited (Couey et al., 2019; Noseda et al., 2024; Durbin et al., 2025; Flores et al., 2026). A rigid post-dose window may therefore create false reassurance.

Multisystem irAEs can initially appear as disconnected complaints. Chest discomfort, palpitations, myalgia, ptosis, dysphagia, dyspnea, and weakness may develop together or sequentially in cardiac–muscle–neuromuscular overlap. Endocrine dysfunction can coexist with gastrointestinal, dermatological, or rheumatological toxicity. The interpretive question is whether a new observation is part of a wider temporal pattern, not whether it can immediately be forced into a single organ category (Wan et al., 2024).

Chronicity changes the relevant outcomes. Permanent endocrine insufficiency, persistent inflammatory arthritis, neurological impairment, pulmonary limitation, dermatological disease, and gastrointestinal sequelae can produce medication burden, rehabilitation needs, work disruption, and repeated specialist care. Acute CTCAE grade alone does not capture long-term function, quality of life, caregiver burden, or survivorship needs (Patrinely et al., 2021; Fletcher and Johnson, 2024).

## 5 Conceptual contribution, terminology, and boundaries

### 5.1 Individual safety alerts and population-level signals

In this review, an individual-level safety alert is a new or worsening symptom, functional change, physiological abnormality, laboratory result, imaging finding, examination finding, or specialist observation that requires verification and contextual assessment. It is not a confirmed irAE, a causal attribution, or a formal pharmacovigilance signal. Formal signal management refers to the population-level process of detection, validation, confirmation, analysis, prioritization, assessment, and recommendation for action (European Medicines Agency, 2017; World Health Organization, 2026). Standardized individual observations may contribute to that process when aggregated across patients and time.

### 5.2 Definition of the immune-related symptom web

The immune-related symptom web is a symptom-centered but not symptom-exclusive active safety-surveillance model situated within the broader pharmacovigilance continuum that organizes dynamic, overlapping, multisystem, and temporally heterogeneous individual-level safety alerts arising during or after ICI exposure. The unit of interest is the evolving relationship among exposure, baseline status, symptom nodes, objective-data nodes, competing explanations, urgency, clinical actions, and subsequent response. The model does not diagnose an irAE, estimate individual risk, grade toxicity autonomously, or determine treatment.

### 5.3 Relationship to existing approaches

Organ-specific guidelines direct investigation and treatment once a syndrome is suspected. CTCAE provides clinician-reported terminology and severity communication. PRO-CTCAE captures selected symptomatic adverse events from the patient perspective but does not establish immune causality (Basch et al., 2014; Bennett et al., 2015; National Cancer Institute, 2017; National Cancer Institute, 2026). ePRO platforms collect repeated reports and can trigger contact. Telephone and oncology triage pathways assign urgency to an individual contact. Statistical symptom networks estimate relationships among symptoms and can identify central or bridging nodes (Papachristou et al., 2019). Conventional pharmacovigilance evaluates aggregated drug-event associations. These approaches are complementary but address different units and phases of safety work. A structured comparison is provided in Supplementary Table S6.

### 5.4 Incremental contribution

**FIGURE 1:**
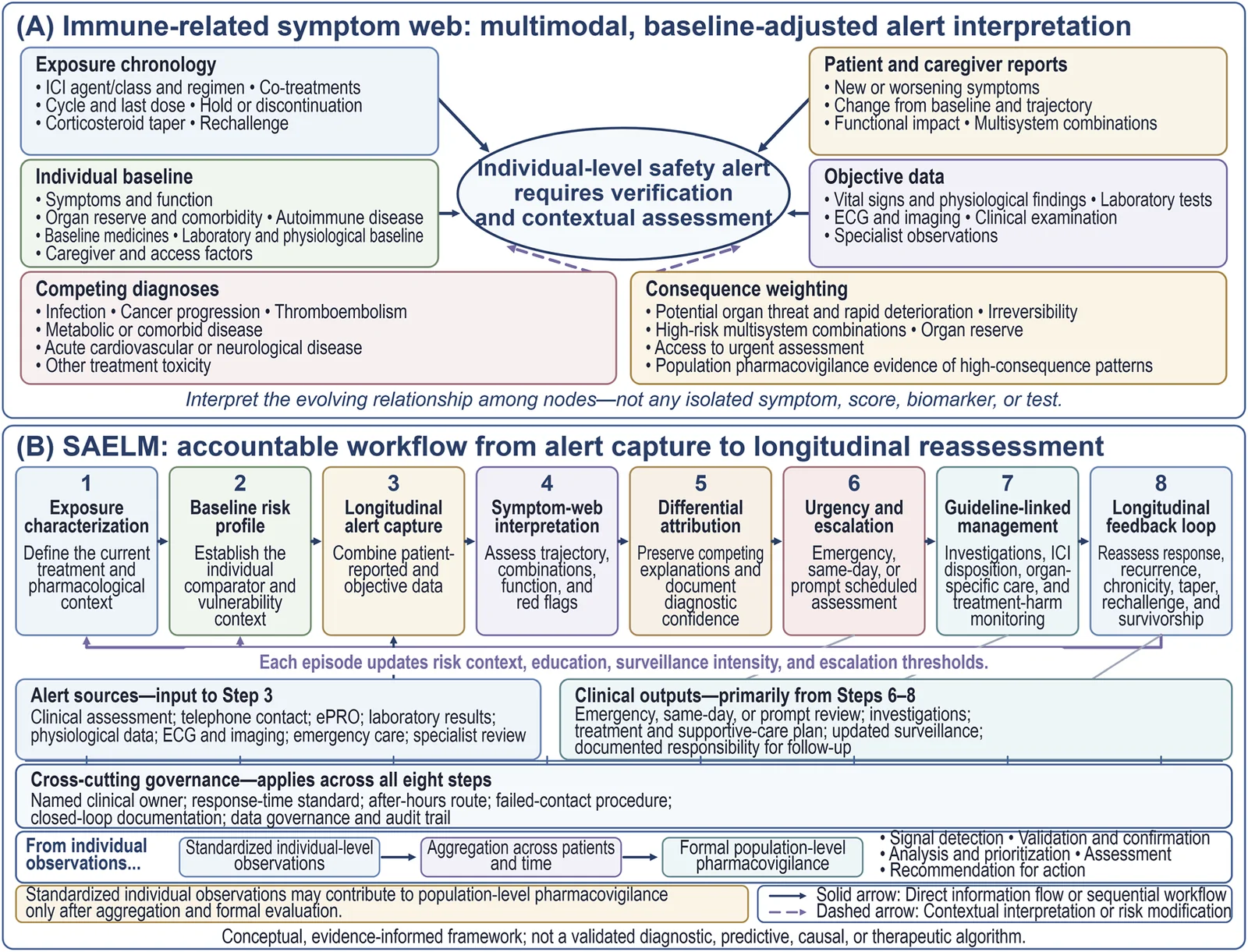
The immune-related symptom web and SAELM framework for longitudinal clinical safety surveillance during and after immune checkpoint inhibitor therapy. **(A)** The immune-related symptom web is a symptom-centered but not symptom-exclusive information model. Exposure chronology and the individual baseline provide the clinical and pharmacological context for patient- and caregiver-reported changes and objective findings, including vital signs, physiological measurements, laboratory results, electrocardiography, imaging, clinical examination, and specialist observations. These inputs generate an individual-level safety alert that requires verification and contextual assessment rather than automatic attribution to immune-mediated toxicity. Competing diagnoses—including infection, cancer progression, thromboembolism, metabolic or comorbid disease, acute cardiovascular or neurological disease, and toxicity from other treatments—and consequence-weighted factors modify interpretation and urgency. The evolving relationship among nodes, rather than any isolated symptom, score, biomarker, or test, is the principal unit of interpretation. **(B)** The Signal‐Attribution‐Escalation‐Longitudinal Management (SAELM) framework translates this information structure into eight sequential and accountable components: exposure characterization, baseline risk profiling, longitudinal alert capture, symptom-web interpretation, differential attribution, urgency assessment and escalation, guideline-linked management, and longitudinal feedback. Patient-reported and objective alert sources feed the alert-capture process; cross-cutting governance—including a named clinical owner, response-time standards, after-hours pathways, failed-contact procedures, closed-loop documentation, and data governance—applies across all eight steps. Clinical outputs arise primarily from urgency assessment, management, and follow-up. Each episode updates the patient’s risk context, education, surveillance intensity, and future escalation thresholds. Standardized individual-level observations may contribute to population-level pharmacovigilance only after aggregation across patients and time and formal signal detection, validation and confirmation, analysis and prioritization, assessment, and recommendation for action. Solid arrows indicate direct information flow or sequential workflow; dashed arrows indicate contextual interpretation or risk modification. The framework is conceptual and evidence-informed and is not a validated diagnostic, predictive, causal, or therapeutic algorithm. ECG, electrocardiography; ePRO, electronic patient-reported outcome; ICI, immune checkpoint inhibitor; SAELM, Signal-Attribution-Escalation-Longitudinal Management.

Baseline-adjusted monitoring, ePRO collection, differential diagnosis, triage, escalation, and closed-loop follow-up are established practices. The proposed contribution is their integration into one longitudinal structure that explicitly connects: (1) exposure chronology and treatment combinations; (2) individual baseline change; (3) symptoms with laboratory, imaging, electrocardiographic, physiological, and examination findings; (4) multisystem combinations; (5) competing diagnoses and changing diagnostic confidence; (6) interruption, corticosteroid tapering, and dechallenge; (7) rechallenge and post-treatment surveillance; and (8) accountable documentation of ownership, response time, action, and follow-up. The symptom web specifies what information should be connected; SAELM specifies how that information should move through clinical care. Figure 1; Tables 1, 2 summarize these relationships.

**TABLE 1:** Minimum data fields, responsibilities, and outputs for the SAELM framework.

SAELM component	Minimum required data	Required output	Accountable role(s)	Evidence status
1. Exposure characterization	ICI target/agent; regimen; setting; co-treatments; cycle and last dose; hold/discontinuation; previous irAE; immunosuppression/taper; rechallenge	Current exposure timeline and monitoring context	Oncology team; pharmacist; treating service	Guideline-supported variables plus author-proposed standardization (Brahmer et al., 2021; Schneider et al., 2021; Haanen et al., 2022; Thompson et al., 2024)
2. Baseline risk profile	Symptoms and function; organ reserve; autoimmune disease; comorbidity; medicines; laboratory/physiological baseline; caregiver and access factors	Individual comparator and personalized escalation threshold	Oncology clinician; nurse/advanced practitioner; pharmacist; patient/caregiver	Evidence-informed practice; proposed minimum dataset (Brahmer et al., 2021; Haanen et al., 2022; Lyles et al., 2021; Rodriguez et al., 2022)
3. Longitudinal alert capture	Patient/caregiver report; clinic or telephone contact; ePRO; laboratory, imaging, physiological, emergency and specialist findings	Time-stamped individual-level safety alert	Named monitoring service with after-hours route	PRO/ePRO and clinical-monitoring evidence; workflow requires validation (Basch et al., 2014; Bennett et al., 2015; Zhang et al., 2022; Lambert et al., 2024)
4. Symptom-web interpretation	Onset; trajectory; functional impact; co-occurring symptoms; objective findings; multisystem pattern; relation to dose, taper and rechallenge	Structured interpretation of stable, improving or accelerating pattern	Trained triage clinician with oncology access	Evidence-informed interpretation; author-proposed integration (Brahmer et al., 2021; Haanen et al., 2022; Lambert et al., 2024; Lai-Kwon et al., 2024)
5. Differential attribution	Immune toxicity; infection; progression; thrombosis; acute cardiovascular/neurological disease; metabolic and comorbid causes; other treatment toxicities	Working differential and documented degree of suspicion	Oncology plus organ specialist/emergency clinician as indicated	Guideline-supported differential diagnosis; proposed documentation structure (Brahmer et al., 2021; Schneider et al., 2021; Haanen et al., 2022; Thompson et al., 2024)
6. Urgency and escalation	Red flags; organ threat; consequence weighting; trajectory; access to urgent assessment	Emergency, same-day or prompt scheduled action; response-time target	Named clinical owner; emergency and specialty pathways	Guideline-supported urgency principles; thresholds require local validation (Mahmood et al., 2018; Salem et al., 2018; Wang et al., 2018; Haanen et al., 2022)
7. Guideline-linked management	Investigations; ICI disposition; immunosuppression; hormone replacement; supportive care; prophylaxis; medication education	Guideline-linked, clinician-authorized plan and monitoring schedule	Oncologist; organ specialist; pharmacist; nursing team	Guideline/consensus supported; organ-specific evidence varies (Brahmer et al., 2021; Schneider et al., 2021; Haanen et al., 2022; Naidoo et al., 2023)
8. Longitudinal feedback loop	Response; diagnostic conclusion; taper; recurrence; steroid dependence; chronicity; residual function; rechallenge; survivorship	Closed-loop resolution and updated baseline, education and surveillance intensity	Multidisciplinary team; primary care/survivorship; patient/caregiver	Evidence-informed follow-up; integrated feedback model is author-proposed (Patrinely et al., 2021; Fletcher and Johnson, 2024; Noseda et al., 2024; Flores et al., 2026)

ICI, immune checkpoint inhibitor; irAE, immune-related adverse event; SAELM, Signal-Attribution-Escalation-Longitudinal Management. Evidence status distinguishes existing guidance or empirical evidence from author-proposed integration; no row constitutes an independently validated treatment or prediction rule.

**TABLE 2:** Tools and data sources for longitudinal detection of ICI-associated toxicity.

Tool or data source	Primary function	Strength	Limitation or misuse to avoid	Implementation and evidence basis
Clinical assessment and CTCAE	Clinician characterization, standardized terminology, severity communication, and linkage to organ-specific guidance	Integrates examination, objective findings, differential diagnosis, and treatment decisions	A grade is not a causal diagnosis; clinician reporting may undercapture patient experience	Training, baseline documentation, organ-threat assessment, and serial reassessment (National Cancer Institute, 2017; Haanen et al., 2022)
PRO-CTCAE or selected symptom items	Direct symptom capture and baseline-adjusted change detection	Captures frequency, severity, interference, amount, or presence from the patient perspective	Not an irAE diagnostic, causality, or autonomous grading tool; does not cover all laboratory-only events	Plain-language item set, defined follow-up, and integration with objective data (Basch et al., 2014; Bennett et al., 2015)
ePRO platform	Repeated between-visit capture, trend display, alert generation, and communication triggering	Can identify trajectories and reduce recall dependence	Non-completion is not wellness; alerts without ownership may be unsafe; digital selection can limit generalizability	Named owner, response standard, after-hours pathway, audit trail, and multimodal alternatives; ICI-specific effectiveness remains uncertain (Zhang et al., 2022; Lambert et al., 2024; Lai-Kwon et al., 2024; Katsura et al., 2026)
Telephone or virtual triage	Focused verification, urgency assignment, safety-netting, and routing	Allows clarification of symptom combinations and immediate questioning	Cannot replace examination or objective assessment; thresholds may vary across settings	Structured protocol, escalation authority, documentation, and closed-loop follow-up; ICI thresholds require validation (Stacey et al., 2013; Piazza and Drury, 2023; Sezgin and Bektas, 2024)
Laboratory, physiological, ECG, and imaging surveillance	Detection of symptomatic or asymptomatic organ abnormalities	Captures toxicities that symptom-only systems may miss	Indiscriminate testing can create false positives; normal early tests may not exclude evolving disease	Regimen- and symptom-directed schedules, trend review, and accountable result ownership (Mahmood et al., 2018; Suresh et al., 2018; Haanen et al., 2022)
Patient-held alert card or treatment summary	Makes current or previous ICI exposure visible across services	Supports emergency, primary-care, and survivorship recognition	Static information may become outdated and does not itself produce follow-up	Clear update responsibility, medication/taper status, contact routes, and red flags (Noseda et al., 2024; Flores et al., 2026)
Spontaneous-report database (e.g., AEMS/FAERS, VigiBase)	Aggregated detection of rare, unexpected, delayed, or disproportionate drug-event reporting patterns	Large scale and useful for hypothesis generation and rare-event characterization	No reliable incidence denominator; under-reporting, duplicates, stimulated reporting, missing verification, and confounding	Complete reports and cautious interpretation (European Medicines Agency, 2017; US Food and Drug Administration, 2026a; World Health Organization, 2026; Noseda et al., 2024)
Active healthcare-data surveillance and registries	Longitudinal population-level safety evaluation with potential denominator information	Can link exposure, outcomes, laboratory data, and follow-up	Confounding, coding error, incomplete capture, and data-governance limitations	Validated phenotypes, data linkage, governance, and analytic transparency (US Food and Drug Administration, 2026b; Wan et al., 2024)

AEMS, adverse event monitoring system; CTCAE, common terminology criteria for adverse events; ECG, electrocardiography; ePRO, electronic patient-reported outcome; FAERS, FDA adverse event reporting system; ICI, immune checkpoint inhibitor; irAE, immune-related adverse event; PRO-CTCAE, Patient-Reported Outcomes version of CTCAE. Tools provide complementary information and should not be conflated.

The originality of the models is therefore integrative, temporal, and testable rather than component-level. Whether this organization improves detection, reduces diagnostic delay, changes management, or improves outcomes remains an empirical question.

## 6 Multimodal alert capture, differential attribution, and risk prioritization

### 6.1 Baseline-adjusted change

Absolute symptom severity can be misleading in patients with cancer, who may begin treatment with fatigue, cough, altered bowel function, pain, neuropathy, rash, endocrine symptoms, or reduced function. Surveillance should ask what is new, worsening, recurrent, accelerating, or functionally different. Baseline documentation should include symptoms, performance and activities, organ reserve, comorbidity, current medicines, selected laboratory and physiological measures, caregiver support, language, health literacy, distance from care, and digital access. Non-completion of a survey is missing information, not evidence of wellness.

### 6.2 Multimodal data

Symptoms often provide the earliest warning between visits, but the framework is not limited to symptoms. Troponin or creatine kinase elevation, liver-test abnormalities, rising creatinine, endocrine abnormalities, hypoxemia, rhythm changes, radiological findings, or an abnormal specialist examination may initiate the same safety workflow in a patient without prominent symptoms (Barroso-Sousa et al., 2018; Cortazar et al., 2016; Delaunay et al., 2019). A useful longitudinal record includes onset, trajectory, functional impact, associated findings, vital signs, laboratory and imaging results, regimen, last dose, co-treatments, recent infection or vaccination, new medicines, previous irAEs, treatment hold, immunosuppressive course, and prior response to intervention.

### 6.3 Dynamic differential attribution

At least five pathways should remain visible at first assessment: immune toxicity; infection; malignancy or paraneoplastic disease; toxicity from chemotherapy, targeted therapy, radiotherapy, corticosteroids, or other medicines; and comorbidity or metabolic disturbance. Thromboembolism and acute cardiovascular or neurological disease warrant explicit parallel consideration. Temporal proximity, improvement after treatment interruption, response to immunosuppression, recurrence during taper, or recurrence after rechallenge may change attribution confidence but do not independently establish causality. The record should document degree of suspicion and the evidence that increases or decreases it.

### 6.4 Consequence-weighted prioritization

Alert priority should reflect not only symptom intensity or event frequency but also the possibility of rapid deterioration, irreversible injury, reported fatal outcomes, high-risk combinations, organ reserve, and access to urgent assessment. Mild dyspnea can accompany early pneumonitis or myocarditis; modest fatigue can reflect adrenal insufficiency; and small changes in strength may precede respiratory compromise. Conversely, severe symptoms may result from non-irAE emergencies.

Population pharmacovigilance can inform which patterns deserve low escalation thresholds, while its limitations must remain explicit. A 2025 analysis of the FDA Adverse Event Reporting System (FAERS) identified 1,326 submitted ICI-associated myocarditis reports and recorded death as an outcome in 37% of those reports (Reeves et al., 2025). A 2026 FAERS study of strict myocarditis–myositis–myasthenia gravis (MMM) overlap recorded death outcomes in 49 of 131 ICI-exposed reports and a median reported onset of 25 days among reports with usable timing (Pacaci et al., 2026). A separate 2026 pharmacovigilance analysis identified heterogeneous fatal-event patterns across ICI toxicities (Yan et al., 2026). These are proportions and patterns within spontaneous reports, not incidence estimates or population case-fatality rates. They nevertheless support consequence-weighted surveillance for cardiac and neuromuscular combinations.

Emergency evaluation is generally warranted for resting dyspnea or hypoxemia; chest pain, syncope, or new arrhythmia symptoms; rapidly progressive weakness, ptosis, dysphagia, respiratory muscle symptoms, seizure, or altered mental status; suspected adrenal crisis or ketoacidosis; severe abdominal pain, bleeding, dehydration, or peritoneal features; acute visual loss; extensive blistering or mucosal skin disease; and systemic instability (Cuzzubbo et al., 2017; Guidon et al., 2021; Sibaud, 2018). Myalgia or weakness combined with chest symptoms, ptosis, dysphagia, or dyspnea should prompt concern for cardiac–muscle–neuromuscular overlap. Urgency should be assigned while the differential remains open and should not await proof of immune causality. Sentinel alerts, potential immune-mediated pathways, competing diagnoses, focused interpretation, and escalation cues are summarized in Supplementary Table S2.

## 7 PRO-CTCAE, ePRO, and digital safety surveillance

PRO-CTCAE was developed to capture symptomatic adverse events directly from patients and complements clinician-reported CTCAE (Basch et al., 2014; Bennett et al., 2015). In ICI care, appropriate functions include symptom capture, baseline-adjusted change detection, trend display, communication triggering, and generation of individual-level safety alerts. It is not an irAE diagnostic test, causality instrument, or autonomous triage algorithm. A low questionnaire score may still be urgent when a symptom is rapidly changing, functionally important, or part of a high-consequence combination.

Randomized trials in broader oncology populations show that electronic symptom monitoring can improve communication and patient-reported outcomes and, in selected settings, reduce acute-care use or produce survival signals (Basch et al., 2017; Basch et al., 2022; Di Maio et al., 2022). ICI-specific programs remain heterogeneous in item sets, frequency, alert thresholds, staffing, response protocols, populations, and outcomes (Lambert et al., 2024). Developmental, co-design, and trial work supports feasibility, but digital eligibility criteria and non-random allocation limit generalizability in some studies (Zhang et al., 2022; da Silva Lopes et al., 2023a; 2023b; Lai-Kwon et al., 2024; Katsura et al., 2026). Current evidence supports prospective testing rather than a claim that ePRO necessarily improves irAE outcomes.

A clinically safe platform should capture a concise high-information item set, allow individual baseline values, display trajectories, and link reports to current ICI and immunosuppressive treatment. Every alert requires a named owner, response-time standard, escalation protocol, after-hours route, failed-contact procedure, documentation standard, and closed-loop follow-up. A dashboard without accountable clinical response is data collection rather than effective safety surveillance.

Reporting frequency should be risk-adapted rather than assumed to be universally weekly or daily. Monitoring may intensify during early combination therapy, after a previous irAE, during corticosteroid tapering, after hospital discharge, or after rechallenge. No universal frequency or minimum item set has been prospectively validated across ICI regimens.

Digital surveillance can widen disparities when smartphone ownership, broadband access, language, health literacy, disability, housing stability, or caregiver support determine who is monitored. Multimodal alternatives should include telephone, paper, interpreter-supported, caregiver-assisted, and community-based options (Lyles et al., 2021; Rodriguez et al., 2022). Completion, response time, escalation, false-negative events, and outcomes should be stratified by access-related variables. Patient-facing text must state whether the platform is continuously monitored, provide emergency instructions, and explain how personal data and alert algorithms are governed.

## 8 The SAELM operational framework

The Signal-Attribution-Escalation-Longitudinal Management (SAELM) framework translates the symptom web into an eight-component multidisciplinary workflow. Within the acronym, Signal denotes structured capture of an individual-level safety alert, not a confirmed population-level pharmacovigilance signal. Table 1 defines the minimum dataset, required output, and accountable roles.

### 8.1 Exposure characterization

Record checkpoint target and agent, monotherapy or combination, treatment setting, concomitant chemotherapy, targeted therapy or radiotherapy, cycle and last dose, current hold or discontinuation, previous irAEs, current corticosteroid or immunosuppressive treatment, taper stage, and rechallenge status. Exposure frames plausibility and monitoring intensity but does not prove attribution.

### 8.2 Baseline risk profile

Document autoimmune disease, organ comorbidities and reserve, baseline symptoms and function, prior radiation fields, previous irAEs, concomitant medicines, frailty, selected laboratory and physiological baselines, caregiver support, language, health literacy, distance from care, and digital access. The profile is updated after major toxicity or treatment change.

### 8.3 Longitudinal alert capture

Combine scheduled clinical assessment, patient and caregiver reports, telephone contacts, ePRO, laboratory abnormalities, physiological data, imaging, emergency presentations, and specialist observations. Capture intensity, change from baseline, trajectory, and functional effect.

### 8.4 Symptom-web interpretation

Assess onset, acceleration, recurrence, co-occurring symptoms, objective abnormalities, multisystem involvement, red flags, and relation to dosing, interruption, tapering, discontinuation, or rechallenge. Identify high-information combinations and determine whether the pattern is stable, improving, or accelerating.

### 8.5 Differential attribution

Keep immune toxicity, infection, cancer progression, thromboembolism, acute cardiovascular or neurological disease, comorbidity, metabolic disturbance, and other treatment toxicities visible until evidence narrows the differential. Document degree of suspicion rather than forcing early certainty.

### 8.6 Urgency and escalation

Assign emergency, same-day, or prompt scheduled evaluation. Activate oncology review, organ-specialist consultation, guideline-directed investigations, emergency referral, and clinician-directed ICI disposition according to organ threat and trajectory rather than a questionnaire score alone.

### 8.7 Guideline-linked management

Link confirmed or strongly suspected syndromes to current organ-specific guidance, supportive treatment, hormone replacement where appropriate, infection prevention, medication education, adherence support, and monitoring of treatment-related harm. The framework records and communicates the plan; prescribing remains a clinician responsibility.

### 8.8 Longitudinal feedback loop

Reassess response, diagnostic confidence, recurrence, steroid dependence, chronicity, residual impairment, treatment resumption, rechallenge, and survivorship needs. Each episode updates the baseline profile, monitoring intensity, education, and future escalation threshold.

### 8.9 Illustrative clinical example

A patient receiving combined CTLA-4 and PD-1 blockade reports new mild exertional dyspnea, proximal weakness, and intermittent ptosis through an ePRO platform. The individual symptom scores are not extreme. The alert is routed to the designated oncology triage service, which reviews the ICI regimen and last dose, baseline function, trajectory, co-occurring chest or bulbar symptoms, current medicines, corticosteroid exposure, available oxygen saturation, and recent laboratory results. Because dyspnea, weakness, and ptosis form a high-consequence cardiac–muscle–neuromuscular pattern, the alert is escalated for urgent assessment rather than managed by the numeric symptom score alone. The record identifies the alert owner, receipt and contact times, working differential, urgency classification, action taken, attendance and results, treatment changes, and the person responsible for follow-up. Subsequent findings update the patient’s baseline and future monitoring intensity. This example is illustrative and does not prescribe a diagnostic test set or treatment regimen.

## 9 Linkage to pharmacological management and stewardship

Management depends on the affected organ, severity, functional threat, diagnostic confidence, comorbidity, treatment intent, available alternatives, and response to initial therapy. Some mild toxicities can be managed with symptom-directed care and close monitoring while ICI treatment continues; moderate events often require temporary interruption; and selected severe, recurrent, or life-threatening events may justify permanent discontinuation. Endocrine replacement-responsive events differ from myocarditis, severe neurological toxicity, or severe cutaneous adverse reactions. Current organ-specific guidelines and institutional protocols should determine treatment (Brahmer et al., 2021; Schneider et al., 2021; Haanen et al., 2022; Thompson et al., 2024).

The central pharmacological question is often whether anti-inflammatory treatment is required before diagnostic certainty is complete. Delay can cause irreversible injury in organ-threatening disease, whereas premature immunosuppression can obscure infection and cause harm. Microbiological studies, imaging, or tissue diagnosis should be obtained before or promptly after immunosuppression when feasible, without delaying emergency treatment.

Corticosteroids remain first-line treatment for many clinically important irAEs, but dose, route, timing, and taper must be organ- and severity-specific. Failure to improve, clinical progression, or inability to taper should prompt diagnostic reconsideration, assessment of adherence and infection, specialist input, and timely organ-directed therapy rather than indefinite corticosteroid escalation. Steroid-refractory and steroid-dependent terminology should be applied with organ-appropriate time frames (Naidoo et al., 2023).

Second-line immunomodulation is syndrome-specific and supported mainly by observational evidence. Infliximab or vedolizumab may be used for selected corticosteroid-refractory or dependent colitis; mycophenolate is commonly considered for immune-mediated hepatitis; and specialist-directed options for pneumonitis, neurological toxicity, myocarditis, or overlap syndromes vary and have limited comparative evidence (Abu-Sbeih et al., 2018; Zou et al., 2021; Salem et al., 2025). Detailed principles are summarized in Supplementary Table S3, which is not a prescribing guide.

Many endocrine irAEs reflect gland destruction and require physiological hormone replacement and acute metabolic stabilization rather than prolonged high-dose immunosuppression. Patients with adrenal insufficiency need emergency identification and sick-day education; those with ICI-associated diabetes require insulin and ketoacidosis prevention; and long-term biochemical follow-up may be necessary.

Medication stewardship is part of longitudinal clinical safety surveillance. Reconciliation should include corticosteroid dose and taper, prophylaxis, endocrine replacement, immunomodulators, antimicrobials, and interacting medicines. Monitoring should address infection, hyperglycemia, hypertension, myopathy, psychiatric effects, osteoporosis, gastrointestinal complications, and adrenal suppression because treatment-related harm can mimic or worsen the original symptom web.

## 10 Rechallenge, chronic toxicity, and post-treatment surveillance

Rechallenge is an individualized risk-benefit decision rather than a fixed algorithm. It may be considered when expected antitumor benefit remains meaningful, alternatives are limited, the initial irAE has resolved or stabilized, and anticipated recurrence risk is acceptable. Relevant factors include the initial organ, severity, reversibility, residual dysfunction, ongoing immunosuppression, prior tumor response, treatment intent, available alternatives, patient preference, and capacity for intensive monitoring (Simonaggio et al., 2019; Dolladille et al., 2020). Severe myocarditis, life-threatening neurological toxicity, or severe cutaneous adverse reactions generally weigh strongly against rechallenge, whereas stable replacement-treated endocrine dysfunction may not.

A rechallenge plan should document rationale, agent or class, reduction from combination to monotherapy where relevant, baseline organ assessment, residual symptoms, immunosuppression, surveillance schedule, and stopping criteria. The monitoring plan should reflect the previous toxicity and update the symptom web if the same or a new toxicity occurs.

Chronic irAEs may require lifelong hormone replacement, disease-modifying antirheumatic treatment, rehabilitation, pulmonary follow-up, neurological support, dermatological care, or ongoing gastrointestinal management. Outcomes should include function, work participation, medication burden, caregiver burden, and quality of life rather than acute grade alone (Patrinely et al., 2021; Fletcher and Johnson, 2024).

Delayed events after discontinuation create a diagnostic hazard because patients, primary-care clinicians, and emergency teams may no longer connect symptoms with previous immunotherapy. Treatment summaries and patient-held or electronic alert records should remain visible across services. Long-term surveillance should cover treatment, holds, immunosuppressive tapering, discontinuation, rechallenge, and survivorship. Its duration and intensity cannot yet be standardized for every regimen, but may reasonably be adapted according to previous severe or multisystem toxicity, persistent symptoms, ongoing immunosuppression, irreversible organ injury, and access to urgent care (Noseda et al., 2024; Durbin et al., 2025; Flores et al., 2026).

## 11 Multidisciplinary implementation, governance, workload, and equity

No single profession can deliver longitudinal ICI safety surveillance across all settings. Medical oncologists integrate cancer benefit, alternatives, treatment interruption, and rechallenge. Organ specialists lead syndrome-specific diagnosis and immunomodulation. Pharmacists support medication reconciliation, prophylaxis, interactions, adherence, and adverse-effect monitoring. Emergency clinicians identify time-critical differentials and stabilize acute illness. Primary-care and survivorship teams maintain vigilance after oncology contact becomes less frequent. Informatics teams design interoperable alert logic, audit trails, and access safeguards. Patients and caregivers provide longitudinal observations.

Oncology nurses are central but not exclusive participants. Nurse-coordinated activities can include baseline education, repeated symptom capture, focused assessment, telephone and digital triage, red-flag recognition, escalation, corticosteroid-taper monitoring, endocrine-replacement education, adherence support, and survivorship coordination. Equivalent functions may be led by pharmacists or advanced practitioners in other systems. Safety depends on training, protocol authority, access to escalation, and closed-loop follow-up rather than professional title alone.

Institutions should define the accountable service for each alert, response-time standards, after-hours coverage, emergency routes, criteria for oncology and specialist consultation, authority to initiate investigations under protocol, and responsibility for closing the loop. A message being sent does not resolve an alert. Documentation should show receipt, contact attempts, assessment, action, attendance, results, treatment change, and follow-up responsibility. Structured communication approaches such as Situation, Background, Assessment, and Recommendation (SBAR) may reduce omissions when adapted to include ICI exposure, last dose, taper status, baseline change, red flags, competing diagnoses, and required response time (Haig et al., 2006).

Education should be repeated at regimen change, initiation or tapering of corticosteroids, treatment hold, rechallenge, hospital discharge, and transition to survivorship. Patients need concrete examples of reportable changes, instructions to report early, and an explanation that toxicities may occur after treatment stops. Emergency and primary-care clinicians should have access to a concise ICI treatment summary.

Workload is a safety outcome. Digital monitoring can shift work from scheduled visits to continuous inbox review. Institutions should track alert volume, response times, contact attempts, clinician time, documentation burden, and after-hours activity. Thresholds optimized only for sensitivity can create alert fatigue; thresholds optimized only for workload can miss evolving toxicity. Human-factors testing, risk-based staffing, and protected response capacity are therefore components of validation.

Equity-sensitive implementation requires alternatives to digital-only surveillance, interpreter access, accessible language, caregiver proxy options where appropriate, disability-compatible interfaces, and proactive outreach for patients with limited access. Reporting, response, escalation, false-negative events, and outcomes should be stratified by age, language, rurality, socioeconomic context, disability, digital access, and health literacy.

## 12 Discussion

### 12.1 Validation agenda and research priorities

Taken together, the proposed contribution is not a new diagnostic taxonomy or treatment algorithm, but a longitudinal information and accountability structure that connects multimodal safety alerts with attribution, consequence-weighted escalation, management, and reassessment. Its potential value therefore depends on whether this organization improves diagnostic safety and clinical response without generating excessive alert burden or unnecessary escalation. These assumptions require staged prospective validation.

Conceptual coherence is not evidence of clinical validity. Before clinical validation, the minimum dataset and candidate alert rules should undergo multidisciplinary and patient co-design across tumor types, regimens, languages, and care settings. Cognitive interviews, content-validity assessment, and burden testing should confirm coverage of high-risk symptoms and objective abnormalities, comprehension, actionability, and feasibility. The prespecified dataset and alert definitions should then be fixed before prospective diagnostic-safety evaluation.

The primary safety outcome should be the severe-event false-negative proportion, defined as the number of independently adjudicated severe or organ-threatening irAEs for which no prespecified alert was generated before clinical diagnosis, divided by the total number of independently adjudicated severe or organ-threatening irAEs. Three key implementation outcomes should accompany it: alert burden per 100 patient-weeks; time from alert receipt to qualified clinical assessment, including compliance with a prespecified response standard; and the proportion of alerts resulting in potentially avoidable urgent escalation. A negative diagnostic work-up alone should not classify an escalation as avoidable; appropriateness should be determined by independent adjudication against prespecified red-flag and urgency criteria. Secondary outcomes can include sensitivity, specificity, predictive values, time from symptom onset to reporting, time to assessment and escalation, patient completion, missingness, clinician trust, and subgroup performance. High-frequency low-acuity symptoms and rare high-consequence syndromes should be reported separately.

The first and most feasible clinical validation study should then be a prospective multicenter observational cohort evaluating feasibility and diagnostic safety before automated risk classification is introduced. Patients starting ICI therapy would undergo standardized baseline assessment and longitudinal capture of patient-reported and objective data using the prespecified minimum dataset. Candidate alerts would be compared with independent clinical adjudication of suspected and confirmed irAEs, with explicit review of hospital presentations and severe events that occurred without a preceding alert.

Only after feasibility and diagnostic safety are established should threshold optimization and comparative implementation studies proceed. Cluster-randomized, stepped-wedge, or hybrid effectiveness-implementation designs could evaluate emergency use, hospitalization, treatment interruption, cumulative corticosteroid exposure, time to second-line immunosuppression, chronic toxicity, function, quality of life, mortality, reach, adoption, fidelity, sustainability, interoperability, and cost. Multicenter collaboration will be required because individual severe irAEs are uncommon.

Longitudinal analytic work can then evaluate symptom networks, latent classes, and trajectories while accounting for time-varying exposure, treatment interruption, death, progression, and competing events. Rechallenge studies require organ-stratified definitions of recovery, recurrence, new irAEs, and ongoing immunosuppression. Post-treatment cohorts should distinguish delayed onset from delayed recognition. Comparative pharmacological studies should use standardized refractory and dependent definitions and address infection, organ recovery, cumulative immunosuppression, cancer outcomes, and treatment variation. Supplementary Table S4 aligns these stages with outcomes.

Candidate biomarkers may eventually refine surveillance, but current genetic, cellular, cytokine, autoantibody, and microbiome markers are not ready to replace clinical and patient-reported monitoring. The effects of corticosteroid timing, intensity, and duration and of organ-directed second-line treatment remain important research priorities.

## 13 Limitations

This review is a narrative synthesis. It did not use duplicate screening, protocol registration, exhaustive database searching, a PRISMA flow diagram, or formal study-level risk-of-bias assessment. Source selection may reflect author judgment and database accessibility. The evidence base is heterogeneous and includes guidelines, observational cohorts, spontaneous-report studies, mechanistic research, and implementation studies with different definitions and ascertainment methods.

Many management recommendations remain consensus-based because randomized evidence for corticosteroid intensity, taper duration, and organ-specific second-line immunomodulation is limited. Guidelines evolve and local protocols vary; this review cannot substitute for the most current organ-specific recommendation. Spontaneous-report analyses cannot establish incidence or causality, and reported death proportions should not be interpreted as population case-fatality rates. Retrospective ePRO associations remain vulnerable to selection and residual confounding.

The immune-related symptom web and SAELM framework have not been validated. No Delphi process, patient co-design, prospective cohort, diagnostic-performance study, or implementation trial was performed for this review. The proposed components may require modification across health systems, professions, languages, and resource settings. Rare but high-consequence toxicities may be poorly represented in general item sets, whereas highly sensitive thresholds may create excessive workload and unnecessary escalation.

The model is symptom-centered but explicitly incorporates objective abnormalities. Even so, no framework based on remote reporting can replace clinical examination, physiological measurement, laboratory monitoring, imaging, and specialist assessment.

## 14 Conclusion

ICI safety surveillance should extend beyond isolated organ-specific reporting toward longitudinal, multimodal, and multisystem assessment across treatment, immunosuppressive tapering, discontinuation, rechallenge, and survivorship. The immune-related symptom web organizes patient-reported and objective changes with exposure history, individual baseline, trajectory, symptom combinations, competing diagnoses, and consequence-weighted urgency. SAELM links this information structure to attribution, escalation, guideline-directed management, accountable documentation, and iterative reassessment.

The frameworks are not validated prediction models, diagnostic tools, causality algorithms, or treatment rules. PRO-CTCAE, ePRO, telephone triage, clinical assessment, laboratory and imaging surveillance, and spontaneous-report data provide different information and should be integrated rather than conflated. The proposed contribution is the longitudinal organization of these elements and the explicit connection between individual clinical safety alerts and potential population-level pharmacovigilance data.

Prospective validation should begin with a multicenter observational cohort focused on false-negative severe events, alert burden, response time, and potentially avoidable escalation. Until safety and utility are demonstrated, the models should be used as evidence-informed structures for communication, data standardization, and research alongside current guideline-directed evaluation.
