# Supplementary material for: The immune-related symptom web in immune checkpoint inhibitor therapy: a longitudinal clinical safety surveillance framework for immune-related adverse events
Source: Front Pharmacol. 2026 Sep 4;17:1942034. doi: 10.3389/fphar.2026.1942034 (PMC13587461; doi:10.3389/fphar.2026.1942034)
Supplement: Supplementary file 1 [file DataSheet1.pdf]

Supplementary Material

Supplementary Methods S1. Representative Search Strategies and Source Selection

The final focused update was completed on 18 July 2026 using PubMed/MEDLINE and the Cochrane Library without a lower date limit. English-language human studies and selected translational or mechanistic studies were eligible at source selection. Because the process was iterative and narrative, a complete run-by-run screening log and record count were not retained. The representative strings below reproduce the principal concept blocks used for the final update and can be rerun; they do not capture every organ-specific refinement and should not be interpreted as a single prespecified or exhaustive systematic-review strategy.

Representative PubMed/MEDLINE string 1—symptom capture and monitoring: ("immune checkpoint inhibitor" OR "immune checkpoint blockade" OR anti-PD-1 OR anti-PD-L1 OR anti-CTLA-4 OR immunotherapy) AND ("immune-related adverse event" OR immunotoxicity OR toxicity) AND (symptom* OR "patient-reported outcome" OR PRO-CTCAE OR ePRO OR "electronic symptom monitoring" OR trajectory OR cluster OR network).

Representative PubMed/MEDLINE string 2—pharmacovigilance and attribution: ("immune checkpoint inhibitor" OR anti-PD-1 OR anti-PD-L1 OR anti-CTLA-4) AND (pharmacovigilance OR FAERS OR AEMS OR VigiBase OR "spontaneous report" OR signal OR causality OR attribution OR surveillance) AND ("adverse event" OR toxicity OR fatal* OR delayed OR multisystem).

Representative PubMed/MEDLINE string 3—management and longitudinal course: ("immune checkpoint inhibitor" OR immunotherapy) AND ("immune-related adverse event" OR immunotoxicity) AND (corticosteroid OR immunosuppression OR refractory OR dependent OR taper OR rechallenge OR chronic OR delayed OR survivorship).

Representative PubMed/MEDLINE string 4—implementation: ("immune checkpoint inhibitor" OR immunotherapy) AND (triage OR telephone OR digital OR eHealth OR implementation OR workflow OR workload OR "alert fatigue" OR equity OR access OR caregiver).

Representative Cochrane Library string: ("immune checkpoint inhibitor" OR "immune checkpoint blockade" OR immunotherapy):ti,ab,kw AND ("immune-related adverse event" OR immunotoxicity OR toxicity):ti,ab,kw AND (symptom* OR "patient-reported outcome" OR PRO-CTCAE OR ePRO OR "electronic monitoring" OR corticosteroid OR taper OR rechallenge OR chronic OR delayed):ti,ab,kw. Organ-specific variants added terms for gastrointestinal, pulmonary, hepatic, endocrine, cardiac, neurological, renal, dermatological, rheumatological, ocular, and hematological toxicities and their principal competing diagnoses.

Targeted sources included current American Society of Clinical Oncology (ASCO), European Society for Medical Oncology (ESMO), Society for Immunotherapy of Cancer (SITC), and National Comprehensive Cancer Network (NCCN) guidance; National Cancer Institute CTCAE and PRO-CTCAE resources; FDA AEMS/FAERS and Sentinel information; EMA signal-management guidance; and WHO pharmacovigilance resources. Backward citation searching was performed from high-priority guidelines, systematic reviews, and seminal studies.

Sources were prioritized according to the type of claim. Guidelines and consensus statements supported clinical management principles; systematic reviews, trials, and cohorts supported comparative or prognostic claims; spontaneous-report studies supported rare-event and reporting-pattern characterization but not incidence; PRO/ePRO studies supported capture and implementation claims; and mechanistic studies were used only to explain clinically relevant patterns. The selection process was iterative, non-exhaustive, and not independently duplicated.

Supplementary Table S1. Pharmacological and Clinical Determinants of ICI-Associated Immunotoxicity

Determinant	Potential influence on toxicity	Evidence limitations	Implications for monitoring
Checkpoint target	CTLA-4, PD-1, and PD-L1 blockade produce overlapping but non-identical population-level patterns. (Khoja et al., 2017; Martins et al., 2019)	Indirect comparisons are confounded by cancer type, dose, follow-up, and event definitions.	Use class to inform, not restrict, symptom questioning and objective surveillance.
Monotherapy versus dual-checkpoint blockade	Dual blockade usually increases overall, severe, and multisystem toxicity. (Wang et al., 2018; Martins et al., 2019)	Risk depends on component dose, sequence, indication, and patient selection.	Consider closer early follow-up and explicit multisystem red-flag education.
Treatment setting	Adjuvant, neoadjuvant, definitive, and metastatic settings differ in duration, competing morbidity, and tolerance for irreversible harm. (Patrinely et al., 2021; Haanen et al., 2022)	Setting is associated with exposure and surveillance intensity; causal effects are difficult to isolate.	Align risk communication, follow-up duration, and survivorship handover with treatment intent.
Concomitant chemotherapy	May add cytopenia, infection, diarrhea, mucositis, fatigue, neuropathy, and organ injury that obscure irAEs. (Haanen et al., 2022; Thompson et al., 2024)	Trial reporting may aggregate adverse events without definitive attribution.	Track cycle timing, blood counts, infection risk, and overlapping toxicity mechanisms.
Concomitant targeted therapy	May amplify hepatic, cutaneous, gastrointestinal, cardiovascular, or pulmonary toxicity depending on target. (Haanen et al., 2022; Thompson et al., 2024)	Regimen-specific evidence cannot be generalized across drug classes.	Maintain a complete medication timeline and evaluate immune and target-specific pathways.
Prior or concurrent radiotherapy	Can reduce organ reserve and create overlapping pulmonary, hepatic, gastrointestinal, neurological, or cutaneous injury. (Haanen et al., 2022; Thompson et al., 2024)	Field, dose, fractionation, and interval vary; radiation recall and immune interactions are incompletely defined.	Document radiation field and dates; lower the threshold for assessment when findings localize to an irradiated area.
Previous irAE	Indicates demonstrated immune susceptibility and may increase recurrence or new-irAE risk during further exposure. (Simonaggio et al., 2019; Dolladille et al., 2020)	Affected by survivor and selection bias; recurrence varies by organ.	Record phenotype, grade, recovery, treatment, and residual dysfunction before resumption or rechallenge.
Pre-existing autoimmune disease	May increase autoimmune flare or de novo irAE risk, although selected patients can receive ICIs. (Abdel-Wahab et al., 2018)	Evidence is largely retrospective and enriched for controlled autoimmune disease.	Establish disease activity, current immunosuppression, specialist plan, and flare indicators.
Organ comorbidity and reserve	May not initiate an irAE but can increase consequence, mimic toxicity, and complicate treatment. (Brahmer et al., 2021; Haanen et al., 2022)	Comorbidity is heterogeneous and inconsistently adjusted in studies.	Document baseline symptoms, physiology, organ tests, and individualized escalation thresholds.
Baseline medicines and polypharmacy	Antibiotics, proton-pump inhibitors, anticoagulants, endocrine agents, nephrotoxins, hepatotoxins, and other drugs can modify risk or differential diagnosis. (Brahmer et al., 2021; Haanen et al., 2022)	Confounding by indication and time-varying exposure is substantial.	Reconcile medicines at baseline and every toxicity episode, including non-prescription products.
Baseline or intercurrent corticosteroids	May mask symptoms, increase infection and metabolic risk, and complicate interpretation of recurrence or adrenal suppression. (Naidoo et al., 2023; Haanen et al., 2022)	Dose, indication, timing, and duration differ; associations with antitumor outcomes are confounded.	Record indication and cumulative course; monitor infection, glucose, muscle strength, mood, bone, and taper-related recurrence.
Treatment interruption or discontinuation	Stops new dosing but does not immediately terminate pharmacological exposure or immune activation. (Couey et al., 2019; Noseda et al., 2024; Flores et al., 2026)	Post-discontinuation ascertainment is often incomplete.	Continue active surveillance and retain ICI exposure in emergency and primary-care records.
Rechallenge	May lead to recurrence of the same irAE, a new irAE, or no further toxicity. (Simonaggio et al., 2019; Dolladille et al., 2020)	Retrospective cohorts reflect clinician selection and heterogeneous definitions.	Use individualized consent, organ-specific recovery criteria, and a personalized monitoring plan.

Determinant	Potential influence on toxicity	Evidence limitations	Implications for monitoring
Patient-level vulnerability and access	Frailty, age-related reserve, transplantation, language, health literacy, distance, finances, disability, and digital access can alter consequences and reporting delay. (Lyles et al., 2021; Rodriguez et al., 2022)	Social and structural factors are under-measured and should not be interpreted as biological susceptibility.	Use multimodal reporting, interpreter access, caregiver support, and proactive outreach; audit equity in response times and outcomes.

Note: CTLA-4, cytotoxic T-lymphocyte antigen 4; ICI, immune checkpoint inhibitor; irAE, immune-related adverse event; PD-1, programmed cell death 1; PD-L1, programmed death ligand 1. These determinants are monitoring variables, not a validated risk score.

Supplementary Table S2. Sentinel Alerts, Potential irAEs, Competing Diagnoses, and Escalation Cues

Sentinel alert	Potential immune-mediated pathways	Important competing diagnoses	Focused interpretation	Escalation cues
New/worsening fatigue, dizziness, or functional decline	Thyroiditis, hypophysitis, adrenal insufficiency, hepatitis, nephritis, myocarditis, inflammatory arthritis, or myositis. (Brahmer et al., 2021; Haanen et al., 2022)	Anemia, infection, progression, dehydration, depression, sleep disturbance, medication effect, steroid withdrawal	Compare with baseline; ask about hypotension, nausea, headache, temperature intolerance, weight, polyuria, taper, weakness, and chest/respiratory symptoms.	Syncope, hypotension, confusion, severe headache, chest pain, dyspnea, profound weakness, or rapid decline.
Cough or dyspnea	Pneumonitis; myocarditis/cardiac failure; myositis or neuromuscular respiratory weakness. (Delaunay et al., 2019; Suresh et al., 2018; Haanen et al., 2022)	Infection, pulmonary embolism, progression, pleural disease, radiation injury, edema, anemia, COPD/asthma	Assess onset, trajectory, rest versus exertion, saturation, fever, sputum, chest pain, radiation history, and thromboembolic risk.	Resting dyspnea, low saturation, chest pain, hemoptysis, syncope, rapid worsening, or weakness/dysphagia overlap.
Chest discomfort, palpitations, or syncope	Myocarditis, pericarditis, arrhythmia, thyroid dysfunction, cardiac–muscle–neuromuscular overlap. (Mahmood et al., 2018; Salem et al., 2018)	Acute coronary syndrome, pulmonary embolism, infection, anemia, dehydration, drug-induced arrhythmia	Record timing, exertional relation, heart rate, myalgia, weakness, ptosis, dysphagia, dyspnea, and recent biomarkers.	Any syncope, persistent chest pain, instability, new arrhythmia symptoms, or cardiac–muscle–neuromuscular overlap.
Diarrhea, increased stool frequency, or abdominal pain	Immune-mediated colitis/enteritis; pancreatic or other gastrointestinal inflammation. (Abu-Sbeih et al., 2018; Zou et al., 2021; Haanen et al., 2022)	Infection, antibiotics, laxatives, diet, chemotherapy, targeted therapy, obstruction, surgery, progression	Quantify stools above baseline, nocturnal symptoms, blood/mucus, fever, pain, hydration, recent antimicrobials, travel, and intake.	Blood, severe/localized pain, peritoneal signs, fever, dehydration, inability to drink, or rapid escalation.
Rash, pruritus, skin pain, or mucosal symptoms	Maculopapular, lichenoid, psoriasiform, bullous, or severe cutaneous immune toxicity. (Sibaud, 2018; Haanen et al., 2022)	Allergic reaction, infection, chemotherapy/targeted-therapy rash, other new medicines	Assess body surface area, spread, pain versus itch, blistering, mucosa, facial edema, fever, and complete medication history.	Skin pain, blistering, mucosal lesions, facial edema, extensive rash, fever, or systemic symptoms.
Headache, visual change, confusion, focal deficit, or seizure	Hypophysitis, meningitis/encephalitis, demyelination, neuropathy, ocular inflammation. (Cuzzubbo et al., 2017; Guidon et al., 2021)	Brain metastasis, stroke, infection, metabolic disturbance, hyperglycemia, primary eye disease	Clarify onset, severity, fever, stiffness, vomiting, hypotension, diplopia, visual loss, weakness, speech, seizure, and endocrine symptoms.	Sudden severe headache, altered mental status, seizure, focal deficit, acute visual loss, hypotension, or meningeal signs.
Myalgia, proximal weakness, ptosis, diplopia, or dysphagia	Myositis, myasthenia-like syndrome, neuropathy, myocarditis overlap. (Mahmood et al., 2018; Guidon et al., 2021; Salem et al., 2018)	Steroid myopathy, electrolyte disorder, deconditioning, statin toxicity, infection, progression	Distinguish pain from weakness; ask about chair rise, neck flexion, breathing, swallowing, ocular symptoms, dark urine, chest pain, and palpitations.	Bulbar/respiratory symptoms, rapidly progressive weakness, ptosis with dysphagia, chest symptoms, arrhythmia, or dark urine.
Jaundice, dark urine, pruritus, or right upper-quadrant discomfort	Hepatitis or cholangitis-like injury. (Brahmer et al., 2021; Haanen et al., 2022)	Biliary obstruction, liver metastasis, viral hepatitis, sepsis, alcohol or other drug-induced injury	Review liver tests, medicines/supplements, alcohol, viral risk, pain, fever, bleeding, and mental status.	Jaundice with confusion, bleeding, severe systemic illness, marked deterioration, or suspected obstruction/sepsis.
Polyuria, polydipsia, nausea, vomiting, or weight loss	ICI-associated diabetes, adrenal insufficiency, hypophysitis, thyroid dysfunction. (Barroso-Sousa et al., 2018; Haanen et al., 2022)	Infection, dehydration, medicines, pre-existing diabetes, gastrointestinal disease	Assess glucose/ketones, intake, urination, pain, hypotension, mental status, and steroid/hormone adherence.	Suspected ketoacidosis, adrenal crisis, hypotension, dehydration, persistent vomiting, or altered mental status.

Sentinel alert	Potential immune-mediated pathways	Important competing diagnoses	Focused interpretation	Escalation cues
Reduced urine output, edema, hematuria, or rising creatinine	Interstitial nephritis, glomerular disease, electrolyte disturbance. (Cortazar et al., 2016; Haanen et al., 2022)	Dehydration, sepsis, obstruction, contrast injury, nephrotoxins, cancer-related kidney injury	Review volume, urine symptoms, medicines, contrast, infection, blood pressure, and laboratory trajectory.	Oliguria/anuria, severe electrolyte disturbance, fluid overload, instability, or rapidly rising creatinine.
Asymptomatic laboratory, physiological, ECG, or imaging abnormality	Hepatitis, nephritis, endocrinopathy, myocarditis, myositis, pneumonitis, hematological toxicity, or other organ injury. (Brahmer et al., 2021; Schneider et al., 2021; Haanen et al., 2022)	Analytical error, pre-existing disease, infection, progression, other treatment toxicity, incidental finding	Verify result and trend; compare with baseline; review exposure, symptoms, medicines, and organ-specific context.	Marked or rapidly worsening abnormality; troponin/ECG change; hypoxemia; severe electrolyte or glucose disturbance; organ-threatening imaging finding.

Note: COPD, chronic obstructive pulmonary disease; ECG, electrocardiography; ICI, immune checkpoint inhibitor; irAE, immune-related adverse event. Alerts are entry points, not diagnoses. Escalation should follow local protocols and should not await proof of immune causality.

Supplementary Table S3. Principles of Pharmacological Management Across Major Organ-Specific irAEs

Organ or syndrome	Initial clinical and ICI considerations	Corticosteroid principle	Selected escalation or steroid-sparing options	Major cautions and longitudinal monitoring
Cross-cutting principles	Assess severity and organ threat; evaluate infection, progression, thrombosis, metabolic disease, and other drug toxicity; ICI continuation, hold, or discontinuation is clinician-directed and organ-specific. (Brahmer et al., 2021; Schneider et al., 2021; Haanen et al., 2022; Thompson et al., 2024)	Use the lowest effective organ-appropriate intensity; severe or rapidly progressive disease may require intravenous or pulse treatment; taper after sustained response.	Choice depends on organ, mechanism, contraindications, evidence, and specialist input.	Screen infection as relevant; monitor glucose, blood pressure, mood, muscle, bone, gastrointestinal and adrenal effects; recurrence during taper may indicate dependent, refractory, or alternative disease.
Gastrointestinal colitis/enteritis	Quantify stool change, hydration, bleeding, pain, fever; test for infection and consider imaging/endoscopy according to severity. (Abu-Sbeih et al., 2018; Zou et al., 2021)	Systemic corticosteroids for clinically significant disease; avoid prolonged ineffective exposure.	Infliximab or vedolizumab are commonly used for selected steroid-refractory/dependent colitis.	Assess infection, tuberculosis/hepatitis risk, perforation, sepsis, and cardiac contraindications; monitor symptoms, nutrition, and recurrence.
Hepatitis or cholangitis-like injury	Review medicines, alcohol, viral hepatitis, obstruction, metastasis, and sepsis; involve hepatology for severe or atypical disease. (Brahmer et al., 2021; Schneider et al., 2021; Haanen et al., 2022)	Systemic corticosteroids for clinically significant immune-mediated hepatitis; tests and response guide taper.	Mycophenolate is commonly considered when refractory; other agents require specialist assessment.	Infliximab is generally avoided in immune-mediated hepatitis; monitor liver function, coagulation, infection, and phenotype-specific response.
Pneumonitis	Assess oxygenation and imaging; exclude infection, embolism, progression, edema, and radiation injury; hold ICI for clinically significant disease. (Delaunay et al., 2019; Suresh et al., 2018)	Systemic corticosteroids; severe hypoxemia or rapid progression requires hospital-level care.	Selected refractory cases may receive specialist-directed mycophenolate, IVIG, infliximab, cyclophosphamide, or other therapy; comparative evidence is weak.	Obtain microbiological evaluation when indicated; monitor oxygen requirement, imaging, pulmonary function, infection, fibrosis, and relapse.
Endocrine toxicity	Urgently treat adrenal crisis, ketoacidosis, severe electrolyte disturbance, or pituitary mass effect; determine deficits. (Barroso-Sousa et al., 2018; Haanen et al., 2022)	High-dose immunosuppression is usually not long-term treatment for stable gland failure; stress-dose corticosteroids are required for adrenal crisis.	Physiological replacement for adrenal, thyroid, gonadal, or other deficits; insulin for ICI-associated diabetes.	Replace glucocorticoids before thyroid hormone when adrenal insufficiency is possible; teach sick-day rules and emergency identification.
Myocarditis, pericarditis, or cardiac toxicity	Emergency oncology and cardiology/cardio-oncology assessment; ECG, biomarkers, imaging, rhythm and hemodynamic monitoring; assess myositis/myasthenia overlap. (Mahmood et al., 2018; Salem et al., 2018, 2025)	Prompt high-intensity corticosteroids are recommended for suspected severe myocarditis while evaluation proceeds.	Refractory or fulminant cases require specialist-directed therapy; abatacept strategies are under prospective evaluation; IVIG/plasma exchange may be relevant in overlap.	Infliximab may worsen moderate-to-severe heart failure; monitor conduction, ventricular function, biomarkers, infection, residual impairment; rechallenge is exceptional.

Organ or syndrome	Initial clinical and ICI considerations	Corticosteroid principle	Selected escalation or steroid-sparing options	Major cautions and longitudinal monitoring
Neurological or neuromuscular toxicity	Urgent neurology assessment for weakness, bulbar/respiratory symptoms, encephalopathy, seizure, neuropathy, or autonomic dysfunction; exclude metastasis, stroke, infection, metabolic and paraneoplastic disease. (Cuzzubbo et al., 2017; Guidon et al., 2021)	Systemic corticosteroids for many inflammatory syndromes; severe presentations often require hospital or intensive-care monitoring.	IVIG or plasma exchange for selected syndromes; rituximab or other agents in specialist-selected refractory disease.	Serial respiratory function and swallowing assessment may be lifesaving; assess cardiac/myositis overlap, infection, IVIG thrombosis risk, and disability.
Nephritis or immune-mediated kidney disease	Review volume, obstruction, sepsis, contrast and nephrotoxins; urinalysis, kidney function and nephrology input; biopsy may clarify atypical/severe cases. (Cortazar et al., 2016)	Systemic corticosteroids for clinically significant immune-mediated nephritis; taper according to renal response.	Mycophenolate or other agents may be considered in refractory disease with nephrology guidance.	Avoid nephrotoxins; monitor creatinine, urine, electrolytes, pressure, infection, and chronic impairment.
Dermatological toxicity	Assess extent, pain, blistering, mucosa, fever and new medicines; urgent dermatology evaluation for severe reactions. (Sibaud, 2018)	Topical therapy may suffice for limited disease; systemic corticosteroids for extensive or severe inflammatory toxicity.	Phenotype-specific steroid-sparing agents; severe bullous or epidermal-necrolysis syndromes require specialist critical care.	Do not delay emergency care for mucosal, bullous, painful, or systemic disease; monitor infection, healing, ocular/genital involvement, and chronic symptoms.
Rheumatological toxicity	Define arthritis, polymyalgia-like disease, myositis, vasculitis, or connective-tissue phenotype; distinguish pain from true weakness. (Haanen et al., 2022)	Local or systemic corticosteroids according to severity; minimize prolonged exposure when chronic disease is likely.	Conventional disease-modifying drugs or selected biologics with rheumatology input.	Monitor function, work participation, bone and infection risk, muscle enzymes when indicated, and cardiac–muscle–neuromuscular overlap.
Hematological toxicity	Urgent hematology evaluation for cytopenia, hemolysis, coagulopathy, aplasia, or hemophagocytic syndromes; exclude infiltration, infection, bleeding, and other drugs. (Brahmer et al., 2021; Haanen et al., 2022)	Corticosteroid use depends on the specific immune hematological syndrome.	IVIG, rituximab, plasma exchange, growth factors, transfusion, or syndrome-specific therapy may be required.	Evidence is limited and phenotypes are heterogeneous; monitor counts, hemolysis/coagulation, infection, thrombosis, transfusion complications, and recurrence.

Note: ECG, electrocardiogram; ICI, immune checkpoint inhibitor; irAE, immune-related adverse event; IVIG, intravenous immunoglobulin. This table summarizes principles from international guidance and selected organ-specific evidence. It is not a prescription; drug choice, dose, contraindications, prophylaxis, duration, and ICI disposition must be individualized.

Supplementary Table S4. Evidence Gaps, Validation Stages, and Clinically Relevant Outcomes

Validation stage or evidence gap	Proposed design	Primary analytic approach	Clinical safety and alert-performance outcomes	Implementation and equity outcomes
Pre-cohort content validity and rule specification	Multidisciplinary and patient co-design across tumor types, regimens, languages, and settings; prespecification of the minimum dataset and candidate alert rules	Cognitive interviews, content-validity indices, consensus methods, and burden testing	Coverage of high-risk symptoms and objective abnormalities; comprehension; actionability; missing domains	Participation diversity; language accessibility; caregiver usability; respondent burden
First-stage validation: feasibility and diagnostic safety	Prospective multicenter observational cohort beginning before ICI initiation; standardized minimum dataset and prespecified alert rules; independent clinical adjudication	Diagnostic-performance analysis with prespecified emphasis on the severe-event false-negative proportion; descriptive alert-yield and response-time analysis	Primary safety outcome: adjudicated severe or organ-threatening irAEs with no prespecified alert before clinical diagnosis divided by all adjudicated severe or organ-threatening irAEs. Secondary: sensitivity, specificity, predictive values, and time to escalation.	Alerts per 100 patient-weeks; time to qualified clinical assessment and compliance with response standards; potentially avoidable urgent escalation; completion and missingness by subgroup
Alert thresholds and frequency	Adaptive comparison of fixed, change-based, red-flag, and risk-adapted schedules after first-stage safety is established	Threshold optimization with explicit weighting of rare high-consequence events	Alert yield, missed emergencies, acute-care use, hospitalization	Alert volume, response-time compliance, adherence, workload, after-hours feasibility.

Validation stage or evidence gap	Proposed design	Primary analytic approach	Clinical safety and alert-performance outcomes	Implementation and equity outcomes
Reproducible phenotypes and trajectories	Prospective longitudinal PRO/ePRO cohorts with adjudicated outcomes	Symptom-network, latent-class, joint or group-based trajectory, and time-varying exposure analyses	Multisystem patterns, onset, recurrence, chronicity, steroid dependence, severe-irAE detection	Missingness by subgroup, data quality, monitoring burden.
Clinical effectiveness of SAELM	Multicenter pragmatic, cluster-randomized, stepped-wedge, or hybrid trial after feasibility validation	Intention-to-treat plus process and mediation analyses	Emergency use, hospitalization, ICU, treatment interruption, corticosteroid exposure, time to second-line therapy, chronic toxicity, function and quality of life	Reach, adoption, fidelity, sustainability, interoperability, cost, patient confidence.
Comparative pharmacological management	Prospective registries and pragmatic organ-specific trials	Target-trial emulation or randomized comparison where feasible; standardized refractory/dependent definitions	Time to response, taper relapse, cumulative immunosuppression, infection, organ recovery, cancer outcomes	Protocol adherence, specialist access, treatment variation, safety across groups.
Chronic and post-treatment irAEs	Extended cohorts beginning before final ICI dose and linked survivorship registries	Multi-state models separating persistence, recurrence, de novo late onset, and delayed recognition	Endocrine replacement, rheumatological/neurological burden, pulmonary impairment, function, late hospitalization	Continuity across oncology and primary care, loss to follow-up, work/financial burden, rural access.
Rechallenge risk-benefit	Prospective organ-stratified cohort with standardized recovery and exposure definitions	Landmark and time-varying analyses; same-irAE and new-irAE competing outcomes	Same-irAE recurrence, new irAE, severity, antitumor benefit, ongoing immunosuppression, alternatives	Shared decision quality, preference, follow-up intensity, access to alternatives.
Equity and health-system value	Hybrid digital/telephone/paper implementation with oversampling of underserved groups and embedded economic evaluation	Interaction and disparity analyses; qualitative evaluation; cost-effectiveness and budget impact	Equal severe-event detection and response; acute-care use; admissions; treatment continuity	Digital access, language, disability, health literacy, caregiver burden, trust, attrition, staffing cost, alert-handling time, travel savings, financial toxicity.

Note: ePRO, electronic patient-reported outcome; ICI, immune checkpoint inhibitor; ICU, intensive care unit; irAE, immune-related adverse event; PRO, patient-reported outcome; SAELM, Signal-Attribution-Escalation-Longitudinal Management. No outcome has yet been demonstrated for the proposed framework. Potentially avoidable urgent escalation should be determined by independent adjudication against prespecified red-flag and urgency criteria; a negative diagnostic work-up alone is insufficient.

Supplementary Table S5. Evidence Provenance of Major Claims and Proposed Framework Elements

Major claim or framework element	Evidence status	Representative supporting sources	What the evidence supports	Principal limitation	Validation requirement
IrAEs may be multisystem, delayed, recurrent, and chronic	Established clinical evidence and consensus	Postow 2018; Martins 2019; Ramos-Casals 2020; Patrinely 2021; Noseda 2024; Flores 2026	Broad phenotype and need for follow-up beyond a single visit or organ	Definitions and follow-up vary; late-event denominators remain limited	Prospective longitudinal and post-treatment cohorts
ICI class and combination influence toxicity patterns	Systematic-review and observational evidence	Khoja 2017; Wang 2018; Martins 2019; Geraud 2021	Population-level tendencies and higher burden with dual blockade	Indirect comparisons are confounded; not an individual prediction	Regimen-specific denominator-based cohorts
Patient-reported symptoms can reveal change not captured by clinicians	Instrument-validation and oncology trial evidence	Basch 2014; Bennett 2015; Basch 2017; Basch 2022	Direct patient perspective and value of repeated symptom capture	Not specific to irAEs; general oncology evidence exceeds ICI-specific evidence	ICI-specific diagnostic-safety and implementation studies
ePRO can support monitoring and communication	Randomized, developmental, scoping, and observational evidence	Zhang 2022; da Silva Lopes 2023a,b; Lambert 2024; Lai-Kwon 2024; Katsura 2026	Feasibility, communication, selected outcome associations	Heterogeneous platforms; digital exclusion; residual confounding	Prospective pragmatic evaluation with workload/equity outcomes
Symptoms or tests do not independently establish immune causality	Guideline and pharmacovigilance principles	Brahmer 2021; Schneider 2021; Haanen 2022; EMA 2017	Need for clinical attribution and differential diagnosis	Diagnostic pathways vary by organ and setting	Independent adjudication in prospective cohorts

Major claim or framework element	Evidence status	Representative supporting sources	What the evidence supports	Principal limitation	Validation requirement
Individual-level alert differs from formal pharmacovigilance signal	Established pharmacovigilance terminology plus author clarification	EMA 2017; WHO 2026; FDA 2026a,b	Formal signals require aggregation, validation, and assessment	Terminology is not uniformly used in clinical monitoring literature	Consistent operational definitions and data standards
Consequence weighting for myocarditis and cardiac–muscle–neuromuscular overlap	Pharmacovigilance and cohort evidence	Salem 2018; Wang 2018; Reeves 2025; Pacaci 2026; Yan 2026	Rare patterns can have high reported severity/fatal outcomes and early reported onset	Spontaneous-report proportions are not incidence or case-fatality rates	Prospective severe-event detection and false-negative analysis
Dynamic differential attribution	Guideline-supported practice	Brahmer 2021; Schneider 2021; Haanen 2022; Thompson 2024	Parallel evaluation of infection, progression, thrombosis, metabolic and other drug causes	Evidence for specific diagnostic sequences is often consensus-based	Organ-specific diagnostic-performance studies
Corticosteroid and second-line treatment principles	Guideline/consensus plus observational organ-specific evidence	Brahmer 2021; Schneider 2021; Haanen 2022; Abu-Sbeih 2018; Zou 2021; Salem 2025	General treatment principles and selected rescue strategies	Comparative trials are limited; recommendations evolve	Pragmatic organ-specific comparative studies
Chronic toxicity, taper recurrence, and rechallenge require longitudinal follow-up	Cohort, review, and consensus evidence	Simonaggio 2019; Dolladille 2020; Patrinely 2021; Fletcher 2024; Flores 2026	Need for ongoing monitoring and individualized rechallenge decisions	Selection bias and inconsistent definitions	Prospective organ-stratified rechallenge and survivorship cohorts
Minimum dataset for the symptom web	Author-proposed integration informed by existing evidence	Guidelines, PRO/ePRO studies, triage literature, pharmacovigilance standards	A testable set of exposure, baseline, alert, attribution, urgency, action, and outcome fields	No formal consensus or content validation	Patient and multidisciplinary co-design, cognitive testing
SAELM workflow and longitudinal feedback loop	Author-proposed operational framework	Evidence-informed synthesis across guidelines, digital monitoring, triage, and implementation studies	Specifies accountable movement from capture to reassessment	No prospective clinical or implementation validation	Multicenter feasibility/diagnostic-safety cohort, then pragmatic trial

Note: The table is a representative evidence-provenance map, not a formal certainty-of-evidence grading exercise. Author-proposed elements require prospective validation.

Supplementary Table S6. Structured Comparison with Existing irAE Monitoring and Pharmacovigilance Approaches

Existing approach	Unit of analysis	Typical starting point	Principal output	Main limitation	What the symptom web/SAELM adds
Organ-specific irAE guidelines (Brahmer et al., 2021; Schneider et al., 2021; Haanen et al., 2022)	Suspected or established organ syndrome	Clinical suspicion of a defined organ toxicity	Diagnostic work-up, grading, treatment, referral, and ICI disposition	Usually begins after organ attribution is suspected	Adds pre-attribution organization and follow-up through tapering, discontinuation, rechallenge, and survivorship
PRO-CTCAE and ePRO systems (Basch et al., 2014; Bennett et al., 2015; Zhang et al., 2022; Lambert et al., 2024)	Repeated patient-reported item or survey	Scheduled or event-triggered patient report	Trend display, communication trigger, or alert	Does not establish causality and may miss objective-only toxicity	Links reports with exposure, objective data, differential attribution, ownership, and documented follow-up
Oncology telephone or digital triage (Stacey et al., 2013; Piazza and Drury, 2023; Sezgin and Bektas, 2024)	Individual patient contact	New symptom or concern requiring clarification	Urgency assignment, safety-netting, and routing	Often focused on one encounter and locally defined thresholds	Embeds triage within a longitudinal record and feedback loop
Statistical symptom-network approaches (Papachristou et al., 2019)	Symptoms as nodes in a cohort-level model	Repeated symptom data suitable for network estimation	Central, bridging, or conditionally related symptoms	Statistical relationships do not provide clinical attribution, ownership, or action	Uses network methods analytically while adding exposure, objective data, attribution, urgency, and action
Conventional population-level pharmacovigilance (European Medicines Agency, 2017; US Food and Drug Administration, 2026a,b; World Health Organization, 2026)	Aggregated drug-event reports or healthcare data	Potential drug-event association across patients	Signal detection, validation, prioritization, assessment, and action	Spontaneous reports lack reliable denominators and do not guide immediate individual triage	Standardizes individual observations that may later contribute to aggregated evaluation without conflating an alert with a formal signal
Immune-related symptom web and SAELM (author-proposed)	Longitudinal individual-level safety alert	Patient-reported or objective change during or after ICI exposure	Capture, attribution, escalation, management linkage, documentation, and reassessment	Conceptual, unvalidated, and dependent on local workflow and resources	Integrates existing components across the treatment and survivorship timeline; complements rather than replaces established approaches

Note: This conceptual comparison does not imply that the proposed symptom web or SAELM replaces existing approaches; abbreviations are defined in the main manuscript.
